# Fructose improves titanium dioxide nanoparticles induced alterations in developmental competence of mouse oocytes

**DOI:** 10.1186/s12917-024-03963-7

**Published:** 2024-04-03

**Authors:** Mohammed A Elmetwally, Amal Helmy, Ahmed Balboula, Mohamed Eladl, Basma Hamed, Samah Lashen, Shaymaa Rezk, Amira Yaseen, Heba Sharawy, Mamdouh Hussien, Samy Zabel, Abdelmonem Montaser, Amal Halawa

**Affiliations:** 1 Department of Theriogenology, Center for Reproductive Biotechnology, Faculty of Veterinary Medicine, Mansoura University, Mansoura, 35516 Egypt; 2 Fertility care center, Obstetrics and Gynecology department, faculty of medicine, Mansoura University, Mansoura, 35516 Egypt; 3 Department of animal science, University of Missouri, Columbia, MO 65211 USA; 4 Center for Reproductive Biotechnology, Faculty of Veterinary Medicine, Mansoura University, Mansoura, 35516 Egypt; 5 Department of Biochemistry and Molecular Biology, Faculty of Veterinary Medicine, Mansoura University, Mansoura, 35516 Egypt; 6 Medical research center, faculty of medicine, Mansoura University, Mansoura, 35516 Egypt; 7 Department of Cytology and Histology, Faculty of Veterinary Medicine, Mansoura University, Mansoura, 35516 Egypt; 8 Department of Theriogenology, Faculty of Veterinary Medicine, Mansoura University, Mansoura, 35516 Egypt; 9 Department of Forensic Medicine and Toxicology, Faculty of Veterinary Medicine, Mansoura University, Mansoura, 35516 Egypt

**Keywords:** Murine oocytes, TiO_2_ NPs, Fructose, Apoptosis, Antioxidant, Oxidative stress

## Abstract

**Aims:**

We investigated the effects of intraperitoneal injections of titanium dioxide nanoparticles (TiO_2_ NPs, 100 mg/kg) for 5 consecutive days on the developmental competence of murine oocytes. Furthermore, study the effects of TiO_2_ NPs on antioxidant and oxidative stress biomarkers, as well as their effects on expression of apoptotic and hypoxia inducing factor-1α (*HIF1A*) protein translation. Moreover, the possible ameliorating effects of intraperitoneal injections of fructose (2.75 mM/ml) was examined.

**Materials and methods:**

Thirty sexually mature (8–12 weeks old; ~ 25 g body weight) female mice were used for the current study. The female mice were assigned randomly to three treatment groups: Group1 (G1) mice were injected intraperitoneal (ip) with deionized water for 5 consecutive days; Group 2 (G2) mice were injected ip with TiO_2_ NPs (100 mg/kg BW) for 5 consecutive days; Group 3 (G3) mice were injected ip with TiO_2_ NPs (100 mg/kg BW + fructose (2.75 mM) for 5 consecutive days.

**Results:**

Nano-titanium significantly decreased expression of GSH, GPx, and NO, expression of MDA and TAC increased. The rates of MI, MII, GVBD and degenerated oocytes were significantly less for nano-titanium treated mice, but the rate of activated oocytes was significantly greater than those in control oocytes. TiO_2_ NPs significantly increased expression of apoptotic genes (*BAX, Caspase 3* and *P53*) and *HIF1A.* Intraperitoneal injection of fructose (2.75 mM/kg) significantly alleviated the detrimental effects of TiO_2_ NPs. Transmission electron microscopy indicated that fructose mitigated adverse effects of TiO2 NPs to alter the cell surface of murine oocytes.

**Conclusion:**

Results of this study suggest that the i/p infusion of fructose for consecutive 5 days enhances development of murine oocytes and decreases toxic effects of TiO_2_ NPs through positive effects on oxidative and antioxidant biomarkers in cumulus-oocyte complexes and effects to inhibit TiO2-induced increases in expression of apoptotic and hypoxia inducing factors.

## Background

Applications of nanotechnology are growing, resulting in an increased release of such products into the environment. Exposure to nanoparticles is associated with health risks [1, 2]. Some nanoparticles may have detrimental effects on reproductive tissues and fetal development because they are smaller and have a larger surface area to volume ratios than the original chemical compounds, allowing them to enter cells and stimulate catalytic and biological activities [3]. Titanium dioxide nanoparticles (TiO_2_ NPs) are natural, highly insoluble, thermally stable and widely manufactured particles [4–6]. They are integrated into numerous industries and products such as paints, plastics, papers, cosmetics, clothing, electronics, toothpastes, food colorants, and sunscreens [7–9]. The potential detrimental impacts of TiO_2_ NPs have been investigated in several studies [10–12]. Both humans and animals can be exposed to nano-titanium via ingestion, inhalation and dermal routes. Oral ingestion is a major route of exposure to TiO_2_ NPs, as a 75 kg adult receives 15-37.5 mg/kg/day from food [13]. Nano-titanium has the ability to pass the biological barriers and accumulate in various organs including the reproductive tract [10, 14]. In vivo exposure of rodents to TiO_2_ NPs results in its transfer to testicles and ovaries which may be detrimental to fertility [15, 16]. From a veterinary perspective, reproduction is one of the main factors keeping the ecosystem in balance. Oocyte maturation in females requires both nuclear and cytoplasmic maturation. Cytoplasmic maturation is the ability of embryos to complete pre-implantation development, whereas nuclear maturation is the resumption of the first meiotic division in oogenesis with extrusion of the first polar body (PB1) [16–18]. In vitro matured (IVM) oocytes have a lower developmental capability than in vivo matured (IVO) oocytes due to insufficient cytoplasmic maturation.

In any culture medium, the energy substrate is one of the most important ingredients for the optimum in-vitro development of oocytes. Glucose is the energy substrate in a single- step medium for IVM of mammalian oocytes [19, 20]. Fructose can also be metabolized via the glycolytic pathway [21]. It is present in bovine reproductive tracts [20, 22, 23] and supports conceptus (embryo/fetus and placenta) development in hamster, bovine, porcine [24], and human embryos [25–27]. Fructose increased total cell numbers in both hamsters and bovine blastocysts [24]. Moreover, the expression of the fructose-transporter gene in bovine embryos emphasizes the embryotrophic role of fructose [26]. Therefore, the objective of the current study was to determine whether intraperitoneal administration of titanium dioxide nanoparticles (TiO2 NPs, 100 mg/kg) for 5 days would affect the ability of murine oocytes to develop normally based on assessment of biomarkers for oxidative stress and antioxidant activity, as well as changes in expression of mRNAs for *BAX, Caspase 3, P53*, and hypoxia inducing factor-1α *(HIF1A).*

## Results

### Effect of intraperitoneal TiO_2_NPs and TiO_2_NPs + fructose on developmental competence of in-vitro matured mouse oocytes

**Fig. 1:**
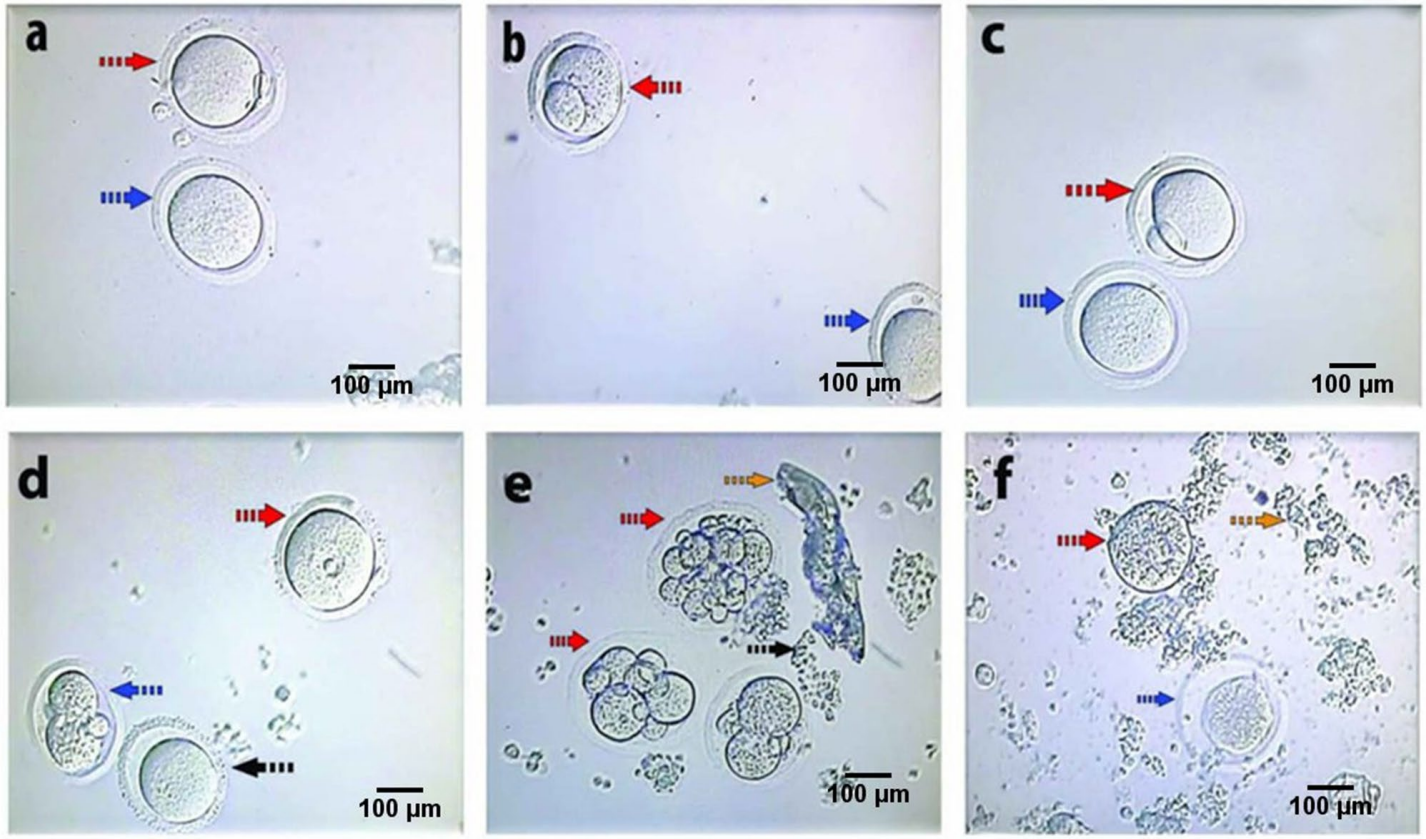
Effects of i/p injection of TiO2 NPs (100 mg), fructose (2.75mM) and TiO2 NPs (100 mg) on the developmental competence of in-vitro matured mouse oocytes:(**a**,**b**) control group (Dotted red arrow: MII with large polar body, Dotted blue arrow: MII with small polar body with large perivitelline space). (**c**,**d**): Group injected with TiO2 NPs (100 mg) + fructose (2.75mM): **c**: Dotted red arrow: MII, Dotted blue arrow: MII with faint polar body. **d**: Dotted red arrow: GV, dotted blue arrow: activated oocyte &Dotted black arrow: MI). (**e**&**f**): Group injected by TiO2 NPs (100 mg) (Dotted red arrow: Activated oocyte, Dotted blue arrow: atretic oocyte

In the current in-vivo study the COCs were exposed to either no treatment (control group, G1: Fig. 1a, b), TiO_2_ NPs (group 2, G2: Fig. 1c, d) or TiO_2_ NPs + Fructose (Group 3, G3: Fig. 1e, f). The percentage of oocytes reached metaphase II (MII) stage was less (*P* < 0.001) for mice in the G2 (18.97 ± 2.52%) and G3 (28.31 ± 3.20%) groups as compared to the control group (34.05 ± 1.57%).

The yield of oocytes that were not able to reach MII (most likely arrested at metaphase I, MI) was greater (*P* < 0.0001) for control and mice injected with TiO_2_ NPs + Fructose than TiO2 NPs treated mice (20.07 ± 1.85%, 22.71 ± 1.16% and 14.84 ± 2.23%; respectively, Table 1).

**Table 1:** Sequences of forward and reverse primers used for qPCR quantitation

Gene	Forward primers	Reverse primers	Accession numbers
BAX	AGACAGGGGCCTTTTTGTTAC	GAGGACTCCAGCCACAAAGAT	XM_015094220.1
Caspase 3	GAATGTCAGCTCGCAATGGTAC	AGTAGTCGCCTCTGAAGAAACTAG	NM_001293722.1
P53	CCCCTGAAGACTGGATAACTGT	GACAGGCACAAACACGAACC	NM_030989.3
HIF 1α	GCAACTAGGAACCCGAACCA	TCGACGTTCGGAACTCATCC	NM_024359.2
B actin	CCCGCGAGTACAACCTTCTT	AACACAGCCTGGATGGCTAC	NM_031144.3

On the other hand, the percent of oocytes undergoing germinal vesicle breakdown (GVBD) was lower for mice in G2 (16.59 ± 0.64%) compared to G3 (18.91 ± 3.93%) and control group (19.34 ± 3.20%) (*p* = 0.0176).

The injection of female mice with TiO_2_NPs (G2) induced an increase (*P* < 0.05) in the percent of degenerated oocytes (24.35 ± 2.23%, *p* < 0.05) as compared with either the control (17.74 ± 2.34%) or the mice receiving a combination of TiO_2_NPs and fructose (G3) (14.11 ± 2.92%) as summarized in Table 1. Further, the percent activated oocytes was greater (*p* < 0.0001) for G2 (25.25 ± 6.05%) and G3 (15.96 ± 4.04%) when compared with the control group (8.80 ± 1.61%).

### Effect of intraperitoneal injections of TiO_2_NPs and TiO_2_NPs + fructose on oxidative stress and concentrations of antioxidant biomarkers for in-vitro matured mice oocytes

As shown in Table 2, the amount of NO in maturation medium with COCs from mice receiving TiO_2_ NPs for 5 consecutive days was reduced (42.89 ± 0.21 mmol/L) when compared to those with COCs from mice in the control (54.39 ± 0.44 mmol/L) and TiO_2_NPs + fructose (51.93 ± 0.42 mmol/L) groups (*p* < 0.0001).

Concentrations of GPx are significantly decreased in COCs from mice in the G2 (386.15 ± 4.6 mmol/L) and G3 (433.52 ± 25.77mmol/L) groups compared to control group (783.77 ± 5.53 mmol/L) (*p* < 0.0001, Table 2).

Concentrations of GSH in in-vitro matured oocytes from mice injected with either TiO_2_ NPs **(**3.13 ± 0.18 mmol/L) **or** TiO_2_ NPs + Fructose (3.50 ± 0.03 mmol/L) groups were less (*p* < 0.0001) than those in in vitro-matured oocytes from mice in the control group (4.74 ± 0.47 mmol/L). Concentrations of MDA in in vitro-matured oocytes from mice in G2 (9.72 ± 0.03 mmol/L) and G3 (9.07 ± 0.11 mmol/L) were greater than those from mice in G1 (7.86 ± 0.04 mmol/L) (*p* < 0.0001, Table 2). Concentrations of TAC in vitro-matured oocytes from mice were greater (*p* < 0.0001) for females in G2 (0.50 ± 0.02 mmol/L) as compared to mice in G1 (0.16 ± 0.001 mmol/L) and G3 (0.25 ± 0.02 mmol/L).

**Table 2:** Effect of I/p injection of fructose (2.75 mM) and TiO_2_ NPS (100 mg/kg BW) on developmental competence of in-vitro matured mice oocytes (In-vivo study)

Treatment	n	MII%	MI%	GVBD%	Degenerated%	Activated%
Control (G1)	168	34.05 ± 1.57^a#^	20.07 ± 1.16^b*^	19.34 ± 3.20^a#^	17.74 ± 2.34^b$^	8.80 ± 1.61^b$^
TiO_2_ NP (G2)	160	18.97 ± 2.52^c$^	14.84 ± 2.23^c$^	16.59 ± 0.64^b$^	24.35 ± 2.23^a#^	25.25 ± 6.05^a#^
TiO_2_ NP + Fructose (G3)	142	28.31 ± 3.20^b*^	22.71 ± 1.85^a*^	18.91 ± 3.93^a#^	14.11 ± 2.92^b*^	15.96 ± 4.04^a#^

Means with different superscripts (a, b, c) are different (*P* < 0.05). Values are presented as means ± SEM

*Abbreviations* MII = metaphase II; MI = metaphase I, GVBD = germinal vesicle break-down, TiO_2_ NP = titanium dioxide nanoparticle

### Transmission electron microscopy (TEM)

**Fig. 2:**
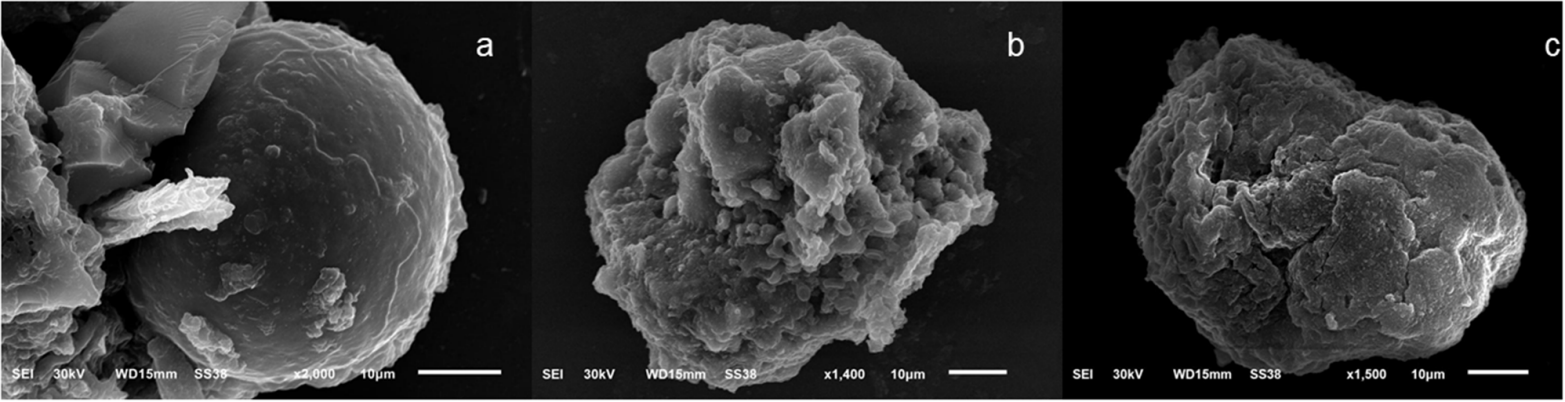
Scanning electron micrographs (SEM) of mature oocytes after being denuded and assigned to either the control or experimental groups. (A) is a control mouse oocyte with a normal round shape and size; (B) oocytes from mice treated with 100 mg/kg TiO_2_Nps i/p have damage to their surface causing loss of round shape and becoming atretic; and (C) exposure of mouse oocytes to 2.75 mM retain a more regular morphology following exposure to toxic effects of TiO_2_ NPs (100 mg/kg)

Scanning electron micrographs of mature mice oocytes after denudation revealed that when compared to those from control mice (Fig. 2a), TiO2 NPs caused damage to the surface of oocytes as evidenced by muliple cytoplasmic projections and irregular shapes and (Fig. 2b). The inclusion of fructose at 2.75 mM ameliorative toxic effects of TiO2 NPs and the morphology of the oocytes was more rounded and regular (Fig. 2c).

### Effects of intraperitoneal injections of TiO_2_NPs and TiO_2_NPs + fructose on the relative expression of mRNAs for *BAX, Caspase 3, P53* and *HIF-1* by COCs from mice

**Fig. 3:**
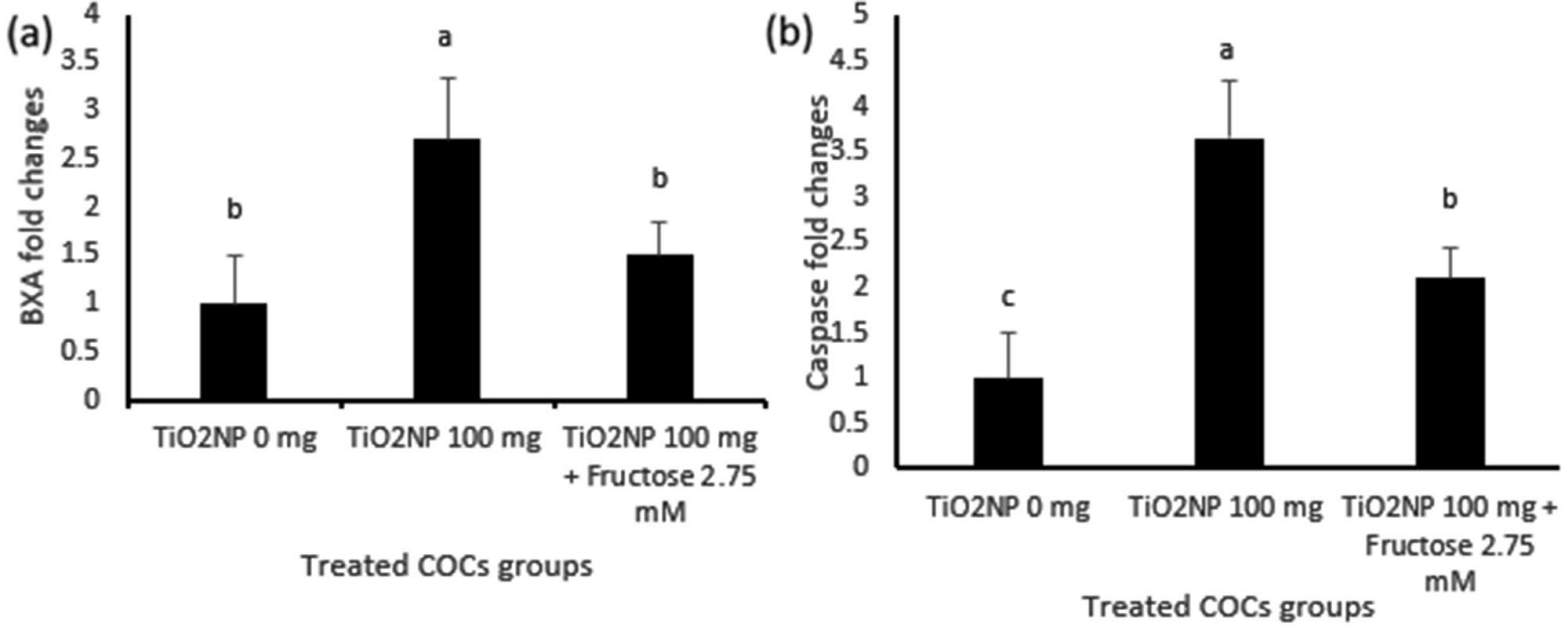
Effects of intraperitoneal injections of TiO_2_ NPs (100 mg/kg) and TiO_2_ NPs (100 mg/kg) + fructose (2.75mM) on the expression of mRNAs for apoptotic genes (**a**) BAX and (**b**) Caspase3, respectively, in in-vitro matured mouse cumulus-oocyte complexes (COCs). The data are presented as means ± SEM. Different letters a, b indicate significant difference (*P* ≤ 0.05) among treatment groups

**Fig. 4:**
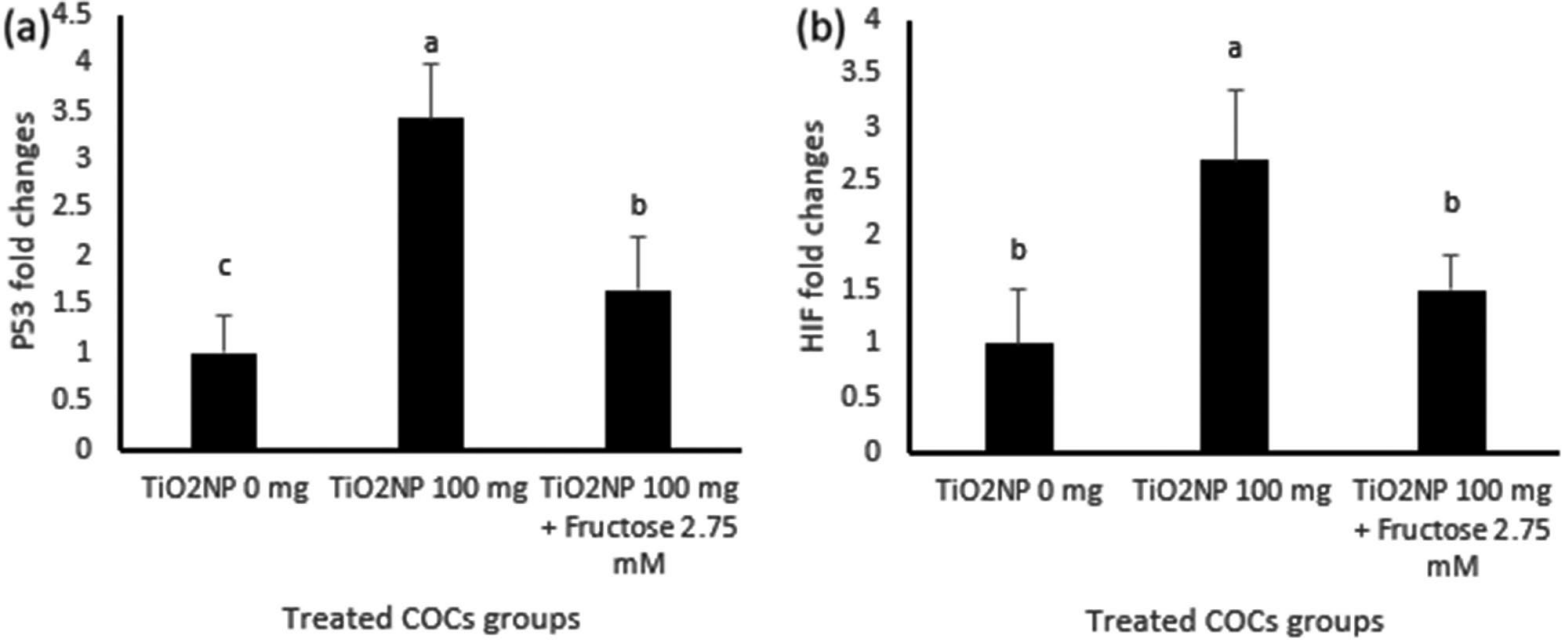
Scanning electron micrographs (SEM) of mature oocytes after being denuded and assigned to either the control or experimental groups. (**A**) is a control mouse oocyte with a normal round shape and size; (**B**) oocytes from mice treated with 100 mg/kg TiO_2_Nps i/p have damage to their surface causing loss of round shape and becoming atretic; and (**C**) exposure of mouse oocytes to 2.75 mM retain a more regular morphology following exposure to toxic effects of TiO_2_ NPs (100 mg/kg)

As shown in Fig. 3, exposure of mice to fructose and TiO2 NPs affected (*p* < 0.05) expression of mRNAs for *BAX*, *Caspase 3, P53* and *HIF-1*α in the recovered COCs. The exposure of mice to TiO2 NPs for 5 successive days led to a 2.7-fold increase in expression of *BAX* mRNA (Fig. 3a) and a 3.7-fold increase in expression of *caspase 3* mRNA (Fig. 3b) as compared to the control group. Likewise, the relative expression of *P53 and HIF1A* mRNAs was 3.4- and 2.7-fold greater in COCs from mice in the TiO2 NPs group as compared to the control group (Fig. 4a and b).

## Discussion

### Effect of I/p injection of fructose (2.75mM) and TiO2NPs (100 mg) on developmental competence of in-vitro matured mouse oocytes

In the current study, we investigated the ameliorative effects of intraperitoneal infusion of fructose after exposure of COCs to TiO2NPs (100 mg). The percentage of oocytes in MII was greater for those from control mice that those for mice treated with TiO2NPs 100 mg and TiO2NPs 100 mg + Fructose 2.75 mM. Also, there was a significant increase in the percentage of oocytes in MII in when mice were treated with TiO2NPs + Fructose compared with oocytes from mice only treated with TiO2NPs. The yield of oocytes in MI was significantly greater for mice injected with TiO2NPs as compared to those from mice in the control group and the group injected with TiO2NPs + fructose. Conversely, the percent of oocytes exhibiting GVBD was less for those from control mice (16.65 ± 0.64%) than for those from mice injected with fructose and/or TiO2NPs. Previous studies with rodents indicate that their exposure to TiO2 nanoparticles in vivo resuts in transfer of TiO2 to the testicles and the ovaries, potentially affecting fertility [15, 28–31]. Because few studies have assessed the potential toxicity of TiO2 nanoparticles on the female reproductive system, the present study determined effects of TiO2 nanoparticles on maturation of mouse oocytes, in vitro fertilization, and the expression of apoptosis-specific genes in blastocysts [15, 30, 32]. Rresults of previous studies and the current study are similar in demonstrating toxic effects of TiO2NPs on developmental competence of mouse oocytes and caused a non-significant decrease in numbers of mature and fertilized oocytes. Ilani et al. (2018) found that numbers of oocytes, 2- and 4-cell embryos, and blastocysts in both experimental groups was lower than for the control group [16].

The injection of mice with TiO2NPs significantly increased the percentage of degenerated oocytes when compared with values for either the control mice or mice receiving a combination of TiO2NPs and fructose. Further, the percentage of activated oocytes was significantly less for those from control mice when compared to those from mice treated with TiO2NPs and TiO2NPs + fructose. Other studies to investigate effects of TiO2 nanoparticles on oocytes at the genomic level found disturbances in embryonic development after fertilization when the embryonic genome is activated, and there were fewer blastocysts that had a greater potential to become apoptotic [16].

### Oxidative stress and concentrations of markers for antioxidants in in-vitro matured mice oocytes: (in-vivo study)

Nano titanium particles disturbed the oxidative/antioxidative equilibrium resulting in a state of oxidative stress in COCs due to lower amounts of NO, GSH and GPx and increased concentrations of MDA. Concentrations of TAC were significantly greater in COCs from mice treated with TiO_2_ NPs than for mice in the control and TiO_2_ NPs + fructose groups. Likewise, TiO_2_ NPs was reported to increase reactive oxidative species (ROS) in mice [15, 33] .

GSH is a cofactor for GPx and its activity is regulated by GST which play a fundamental role in maintaining cellular redox status and protecting cells from oxidative damage. MDA, GPx and TAC are involved in interactions between ROS and lipids that increase in response to increases in ROS [34]. Thus, MDA,TAC and GPX are commonly used as ROS-related biomarkers [28, 30]. In the present study, we analyzed the pelleted COCs after resuspension in BPS from mice in the various treatment groups for MDA,TAC and GPx and found their concentrations to be greater for those from mice in the TiO2NP group than for control mice. Those results support results from a previous study [33]. Also, NO and GSH were less abundant in in COCs collected from mice treated with TiO2NP as compared to values for COCs from control mice. Those results are similar to those from meta analyses of in vivo studies, using the rat as a model animal and published between 2013 and 2022, investigating the protective roles of antioxidants for toxicity induced by nanoparticles at daily doses of 5, 100, and 200 mg orally or intravenously for 1 to 8 weeks. The toxic effects of TiO2NPs exposure may be involve both direct and indirect pathways [35]. In direct pathway, TiO2NPs enter the blood after oral exposure and is then distributed to different tissues such as liver, spleen, kidney, lung [36] and ovary [30]. In current study, it seems that the accumulation of this nanoparticle in the ovaries was toxic based on results of our biochemical and morphological evaluations. In an indirect pathway, oral administration of TiO2NPs affects the ovary by increasing ROS levels and inducing inflammatory responses [37]. However, the role of fructose in vivo remains controversial, since acute temporary application of fructose may protect yeast, as well as animal tissues against exogenous oxidative stress. The review by Semchyshyn suggests the involvement of reactive carbonyl and oxygen species in both the cytotoxic and defensive effects of fructose [38].

In the current in-vivo study, the percentage of oocytes in MII was significantly greater for those from mice treated with TiO2NPs + fructose than for mice treated with TiO2NPs. Also, there is a decrease in the percentages of oocytes in MI, those undergoing GVBD and those degenerating from mice treated with TiO2NPs + fructose as compared to mice treated with TiO2NPs. Our previous results showed the protective and ameliorating role of fructose against adverse effect of TiO2NPs in the developmental competence of mice oocytes [39]. The results were also in agreement with evidence that nonenzymatic reactions of fructose and higher production of reactive carbonyls (RCS) and oxygen species (ROS) play a dual role in vivo regarding reactive species with fructose having both cytotoxic and defensive effects [38].

Although a long-term consumption of excessive fructose may have adverse side effects, its acute temporary ingestion can be beneficial under some circumstances. For example, short-term application of fructose protectS astroglial C6 cells against peroxide-induced stress [40]. In the present study, there was an increase in NO,GSH,MDA and TAC in culture medium, but a decrease in GPx in culture medium for COCs from mice treated with TiO2NPs + fructose than those from mice treated with only TiO2NPs. These findings prompted us to propose an additional explanation for the protective effect of fructose. Fructose induces a mild carbonyl/oxidative stress-stimulating cellular defensive mechanism responsible for cell survival under lethal stress [41]. The last mechanism can be posited from results of in vitro and in vivo studies indicating that fructose is a potent glycoxidation agent. Our findings indicate a protective role of fructose against oxidative stress caused by TiO2NPs and this is agreement with Frenzel et al., who reported that fructose inhibited apoptosis induced by reoxygenation of rat hepatocytes by decreasing ROS [42] and with MacAllister et al., who documented that fructose protects rat hepatocytes against exogenous oxidative stress [43].

Fructose and its phosphorylated derivatives such as fructose-1,6-bisphosphate have significantly greater antioxidant capacities against ROS than other carbohydrates [44] suggesting that acute infusion or ingestion of fructose could benefit in the cytoprotective therapy of disorders related to oxidative stress [42]. According to the theory of homeostasis, steady-state concentrations of oxidants and antioxidants are maintained within a limited range [45]. In contrast to strong antioxidants, fructose and its phosphorylated derivatives (e.g., fructose-1,6-bisphosphate) are important energy substrates. The beneficial effects of fructose-1,6-bisphosphate have been documented in different tissues, including the heart, liver, kidney, brain, and small intestine [46]. The cytoprotective mechanisms underlying fructose-1,6-bisphosphate are believed to be involved in its intervention in the glycolytic pathway, as a metabolic regulator or substrate, as well as an agent modifying ion permeability of cell membrane transporters.

An obvious question is what mechanism(s) is responsible for the protective effect of the short-term application of fructose? Several mechanisms may be responsible for the protective effects of fructose: [1] iron binding and prevention of the Fenton reaction [40]; [2] stabilization of the glutathione pool in the cell [42]; [3] upregulation of the pentose phosphate pathway producing NADPH [44] and [4] production of fructose-1,6-bisphosphate that mediates cytoprotective and antioxidant mechanisms [40, 46].

To our knowledge, there are no published results concerning effects of adding TiO2NPs + fructose to IVM media on developmental competence of female mice oocytes in-vitro or injecting TiO2NPs + fructose on developmental competence of female mice oocytes in-vivo. Thus, we are not able to compare findings obtained from the present study with results of other studies. Our study is not without limitation. We did not measure levels of other sex hormones (LH and FSH) and, we used only one dose of TiO2NP and one dose of fructose for 5 concessive days. Therefore, we propose to apply lower doses of exposure in future studies as results of the current study indicate that 1.25mM of fructose in IVM media improve developmental competence of mammalian oocytes.

Toxic effects of TiO2NPs in IVM media and i/p injection in-vivo may affect developmental competence of mammalian oocytes, fertility and damage to ovarian tissue that may lead to ovarian dysfunction associated with lipid peroxidation and an imbalance in sex hormones. Therefore, results of the present study provide useful information regarding the risk of TiO2NPs in young females, especially during childbearing years. Fructose can ameliorate toxic effects of TiO2NPs in-vitro and in-vivo to improve the developmental competency of mammalian oocytes.

The toxic effects of the nanoparticles have been studied using male rather than female animal models [47]. Oocytes, in contrast to male gametes, are not protected by a blood-testis barrier making them vulnerable to toxins that may adversely affect fertility. Furthermore, vascular permeability increases during the last stages of follicular development and ovulation to allow even large components of serum to enter [48]. Those components, along with products released from cumulus cells and oocytes enable the formation of follicular fluid that fills the antrum of ovarian follicles. Due to this physiological process, potentially harmful chemicals in blood may affect oocytes [49, 50]. The current study investigated, for the first time, effects of TiO2NPs on the integrity of murine oocytes via EM imaging. The exposure of murine oocytes to TiO2NPs at doses of 50 mg or 100 mg caused many abnormal cytoplasmic projections and asymmetrical forms on their surface while fructose at 1.25 and 2.75 mM ameolrated toxic effects of TiO2NPs. Moreover, the in vivo exposure of oocytes to 50 and 100 mg TiO2NPs caused full destruction to the oocyte surface resulting in the loss of the oocyte’s spherical shape and induction of atresia. Previous studies showed that the in-vitro exposure of murine oocytes to cerium dioxide nanoparticles had genotoxic effects on both oocytes and cells of the follicle [51]. Another study showed that oral treatment of female mice with titanium dioxide nanoparticles (TiO2NPs) disrupted ovarian gene expression and caused a hormonal imbalance which lowered rates of conception [52]. Similarly, exposure of murine oocytes in-vitro to TiO2, ZnO, and SiO2-coated ZnO nanoparticles interfere with expansion of cumulus cells in-vitro at both morphological and molecular levels [47]. In pigs, 3 mM fructose improved oocyte maturation and embryonic development after activation of parthenogenesis in vitro [53].

The genotoxic effects of i/p infusion of TiO2NPs on murine COCs were investigated for the first time in the present study. The effects of fructose and TiO2NPs on expression of *BAX, Caspase 3, P53*, and *HIF1A* mRNAs in mice COCs were significant. COCs up-regulated expression of the apoptotic and hypoxia-inducing mRNAs studied after 5 days of exposure to 100 mg TiTO2NPS. The expression of *caspase 3* mRNA showed the greatest fold-change. Furthermore, 2.75 mM fructose reduces adverse effects of TiTO2NPS. In COCs from mice, the ameliorative impact of fructose was evident as significant decreases in *BAX, Caspase 3, P53*, and *HIF1A* mRNA expression. Those results are similar to those from a study of rats assessing effects of TiTO2NPS on the expression of apoptotic markers in blastocysts [16]. In addition to decreased numbers of 2-cell and 4-cell embryos and blastocysts in the TiTO2NPS treated rats, the expression of *BAX* and *caspase3* was significantly increased and expression of *BCL* xl genes was significantly decreased by treatment of rats by TiO2NPs [16]. The female mice exposed to TiO2 nanoparticles had nanoparticles deposited in the ovaries, histological changes in ovarian granulosa cells, increased numbers of atretic follicles, changes in concentrations of sex hormones, and significant reductions in ovarian weight and fertility [15, 30, 52]. Other studies investigating effects of titanium dioxide on preantral ovarian follicles revealed that rates of formation of antral follicles and maturation of oocytes decreased significantly with increasing concentrations of TiO2.

The present study investigated, for the first time, the ameliorative effects of fructose on genotoxic effects of TiO2NPS and found that the i/p injection of mice with fructose decreased the genotoxic effects of TiO2NPS on murine COCs by decreasing expression of mRNAs associated with apoptosis. In pigs, fructose supplementation significantly increased germinal vesicle breakdown (GVBD), maturation to metaphase II (MII), sperm penetration, and development of pronuclei in oocytes (Reference). The incorporation and oxidation of 14 C-methionine in oocytes increased in to a greater extent in response to fructose supplementation as compared to glucose supplementation. The incorporation and oxidation rates of 14 C-fructose were substantially greater [26].

## Conclusion

In conclusion, TiO2 nanoparticles at 50 and 100 mg/kg via i/p in mice have detrimental effects on COCs through the induction of apoptosis genes in COCs and fructose is effective in ameliorating detrimental effects of TiO2NPs.

## Materials and methods

This study was conducted at the Theriogenology department, Faculty of Veterinary Medicine, Mansoura University, Egypt, with an approved animal use protocol (PhD/29) in accordance with the Guiding Principles for the Care and Use of Research Animals. The preparation and characterization of TiO_2_ NPs was described in our previous publication [11].

Fructose (≥ 99%: CAS number 57-48-7) was purchased from Sigma-Aldrich Co. LLC (St. Louis, MO, U.S.A.).

### Animals, treatments and experimental design

Thirty sexually mature (8–12 weeks old; ~ 25 g body weight) female mice were used for the current study were purchased from the Medical Experimental Research Center (MERC) of Mansoura University, Egypt. The mice were kept in temperature-controlled conditions under a 12 h light/dark cycle and allowed to freely access water and feed ad libitum in the Medical Experimental Research Center (MERC) of Mansoura University. To induce superovulation, each female mouse received an intraperitoneal injection of 10 IU of pregnant mare serum gonadotropin (PMSG, Gonaser®, HIPRA, Spain) 48 h prior to euthanization by cervical dislocation of mice according to previous studies [54, 55]. This experiment was designed to assess the effects of i/p injections of TiO_2_ NPs (100 mg/kg BW) and TiO_2_NPs (100 mg/kg BW) + Fructose (2.75mM) on maturation of immature oocytes, intracellular oxidative stress (MDA and NO) and antioxidant biomarkers (GPX, GSH, and TAC) of COCs. The female mice were divided into three groups: Group1 (G1) mice received deionized water and collected oocytes were cultured in global® total medium (control group); Group 2 (G2) mice received TiO_2_NPs at 100 mg/kg BW by i/p injection for 5 consecutive days, and the collected oocytes were cultured in global® total medium; and Group 3 (G3) received TiO_2_ NPs at 100 mg/kg BW + 2.75 mM fructose by i/p injection for 5 consecutive days [26], and the collected oocytes were cultured in global® total medium.

### Oocytes recovery after collection of ovaries

The ovaries were freed of fat and rinsed in warm sterile physiological saline (0.9% NaCl) to remove blood [56]. After that, the ovaries were put in a sterile 60 mm Petri dish with G-MOPS™ Plus Medium (Vitrolife, Sweden) to recover the oocytes. The release of cumulus-oocyte complexes (COCs) from the ovaries was achieved by gently puncturing the antral follicles with a sterile insulin syringe needle under a SZ61 zoom stereo microscope (Olympus, Japan ,2016) [57]. COCs released were identified and sorted according to their morphological characteristics. Only high-quality COCs of oocytes that showed uniformly granular ooplasm and were surrounded by compact multi-layers of follicular cells were used [57].

### Fructose preparation

For preparing 2.75 mM fructose solution, fructose particles were suspended in deionized water via ultrasonication for 15 min just before administration. The stock solution was immediately aliquoted, protected from light, and stored in a refrigerator (4 ℃ ) till the day of use. This concentration was chosen based on results of a previous study [26].

### In-vitro maturation of mouse cumulus–oocyte complexes (COCs)

Immature female mouse oocytes were matured in vitro in pre-equilibrated medium **(Global® total®, USA)** as described previously [58]. Briefly, COCs were washed three times in 50 µl droplets of **G-MOPS™ plus** medium (Vitrolife, Sweden) and then another three washes in 50 µl droplets of Global total medium. As described previously [59], the COCs were assigned randomly for culture in groups of 20–25 oocytes in a 50 µl droplet of prewarmed IVM medium under sterilized mineral oil for 17 h at 37 °C under and atmosphere of 5% CO_**2**_ in air with 95% humidity.

### Assessment of mature (MII), MI, GVBD, activated and degenerated oocytes rates

After 17 h of their incubation in Global total medium, oocytes were denuded from surrounding cumulus cells by gentle pipetting as shown in Fig. 1. Maturation rates were based on breakdown of the germinal vesicle with formation of the MII spindle and/or extrusion of the first polar body. The rates of MI, GVBD, degeneration and activation of oocytes was calculated by dividing total number in each category by total number of cultured oocytes [60].

### Biochemical analysis

For biochemical analyses, four samples were analyzed per group. Each sample included 20–25 oocytes in 100 µl of IVM medium. The oocytes were pelleted by centrifugation at 3,000 rpm for 10 min at 4 ºC. The cell pellets were gently rinsed twice with 500 µl of cold phosphate-buffered saline (PBS). The pellets were disrupted in 800 µl of lysis buffer (50 mM sodium phosphate, 300 mM NaCl, pH = 8.0) and three cycles of repeated freezing and thawing followed by vortexing. Lysed oocytes were centrifuged at 12,000 rpm for 15 min at 4 ºC and the supernatant decanted and used for biochemical analyses.

### Measurement of intracellular oxidative stress and biomarkers of antioxidative state

Concentrations to the intracellular oxidative biomarkers, NO and MDA [61], within in-vitro matured oocytes were determined colorimetrically using commercially available kits (Biodiagnostics, Egypt) per manufacturer’s instructions. Concentrations of intracellular antioxidant levels, TAC [62], GSH [63] and GPX [63, 64], within in-vitro matured oocytes were determined using colorimetric assay kits (Biodiagnostics, Cairo, Egypt) per manufacturer’s instructions.

### Transmission electron microscopy (TEM)

The oocyte recovery and collection were performed blinded to remove any biases in selection for TEM. All specimens were immediately immersed in 2.5% paraformaldehyde-glutaraldehyde in 0.1 M sodium cacodylate buffer, pH 7.4, and stored at 4 °C until processed. The samples were further centrifuged at 5000 xg and pellets were washed three times with sodium cacodylate buffer. They were post-fixed in 1% osmium tetroxide in a sodium cacodylate buffer and then dehydrated in an ascending series of ethanol and embedded in Super’s resin. Ultra-thin sections were stained with uranyl acetate and lead citrate and examined by transmission electron microscope (JEM-1011, Jeol Co., Tokyo, Japan) at the EM-Unit, Faculty of Agriculture, Mansoura University.

### Gene expression analysis

All primers were designed using Primer-BLAST software (http://www.ncbi.nlm.nih.gov/tools/primer-blast/). These primers include those for *Bax, caspase 3, P53* and *HIF1A*, genes associated with apoptosis in mouse oocytes. The sequences of forward and reverse primers are provided in Table 3. Extraction of mRNA from oocytes was performed using Trizol reagent (Thermo Fisher Scientific, UK) according to the manufacturer’s instructions [65]. One microgram of extracted RNA was used to prepare cDNA using the Hisenscript™ cDNA synthesis kit (Intronbio, South Korea). The newly formed cDNA strand was used as a template for gene expression analyses using primer pairs for *Bax, caspase 3, P53* and *HIF1A*. PCR was conducted using the Pikoreal real-time PCR system (Thermo Scientific, Lituania). PCR cycling conditions were as follows: initial denaturation at 94 °C for 9 min followed by 35 cycles of denaturation at 94 °C for 1 min, annealing 55 °C for 40 s, and elongation 72 °C for 1 min, then the PCR cycling was terminated by a final elongation step at 72 °C for 9 min. Gene expression analyses used B-actin as the housekeeping gene following the method of Livak and Schmittgen, 2001 [65], using the 2-AAct method for calculation of fold-changes in expression relative to the control group.

**Table 3:** Effect of i/p injection of fructose (2.75 mM) and TiO_2_ NPS (100 mg/Kg) on oxidative stress and antioxidant biomarkers of in-vitro matured mice oocytes: (In-vivo study)

Treatment	n	NO (mmol/L)	GPx (mmol/L)	GSH (mmol/L)	MDA (mmol/L)	TAC (mmol/L)
Control (G1)	168	54.39 ± 0.44^a^	386.15 ± 4.61^c^	4.74 ± 0.47^a^	7.86 ± 0.04^c^	0.16 ± 0.001^c^
TiO_2_ NP (G2)	160	42.89 ± 0.21^c^	783.77 ± 5.53^a^	3.13 ± 0.18^c^	9.72 ± 0.03^a^	0.50 ± 0.02^a^
TiO_2_ NP + Fructose (G3)	142	51.93 ± 0.42^b^	433.52 ± 25.77^b^	3.50 ± 0.03^b^	9.07 ± 0.11^b^	0.25 ± 0.02^b^

Means with different superscripts (a, b, c, d) are different (*p* < 0.05). Values are presented as means ± SEM

*Abbreviations* n = number of oocytes; NO = nitric oxide; GPx = Glutathione peroxidase (GPx); GSH = Glutathione; MDA = Malondialdehyde acetate; TAC = Total antioxidant capacity, TiO_2_NP = titanium dioxide nanoparticles

### Statistical analysis

Statistical analyses were according to those published previously by Elmetwally et al. [66, 67]. The normality of quantitative data (developmental competence, antioxidant and oxidative stress biomarkers, and mRNAs for apoptotic genes) was determined using normal probability plots and the Kolmogorov-Smirnov test generated by SAS’s UNIVARIATE procedure. The experimental data are expressed and means their standard errors (SEM). A one-way analysis of variance (ANOVA) was used to determine the effects of fructose and titanium dioxide nanoparticles on oocyte maturation followed by Duncan’s multiple comparison test. The SAS® program was used for statistical analyses (version 9.2, SAS Institute, Cary, NC, USA). *P* ≤ 0.05 was considered significant.

## Data Availability

The datasets used and analysed during the current study are available from the corresponding author on reasonable request.

## Abbreviations

CAT: Catalase
COCs: Cumulus-Oocyte Complexes
FSH: Follicular stimulating hormone
GPx: Glutathione peroxidase
GSH: Glutathione
GVBD: GV breakdown
I/P: Intra peritoneal injection
IVM: In vitro maturation
LH: Lutilizing Hormone
MDA: Malondialdehyde
mg: Milligram
MII: Metaphase of the second meiosis
mM: Millimole
NO: Nitric oxide
NPs: Nanoparticles
PBS: Phosphate-buffered saline
ROS: Reactive oxygen species
SEM: Standard Error of Mean
TAC: Total antioxidant capacity
TEM: Transmission electron microscopy
TiO2: Titanium dioxide
TiO2 NPs: Titanium dioxide nanoparticles
